# ERBB3 Promotes Malignant Behaviors of Endometrial Cancer Cells with Involvement of the Ras-ERK/MAPK Signaling Pathway

**DOI:** 10.3390/cancers18111765

**Published:** 2026-05-28

**Authors:** Yuanlin Liu, Hu Li, Xiaofeng Li, Mingyuan Li, Yiran Li

**Affiliations:** 1 Department of Gynecology, Shanghai First Maternity and Infant Hospital, School of Medicine, Tongji University, Shanghai 200092, China; 2280292@tongji.edu.cn (Y.L.); 2511148@tongji.edu.cn (H.L.); 1632110@tongji.edu.cn (X.L.); 2 Shanghai Key Laboratory of Maternal and Fetal Medicine, Shanghai Institute of Maternal Fetal Medicine and Gynecologic Oncology, Shanghai First Maternity and Infant Hospital, School of Medicine, Tongji University, Shanghai 200092, China; 3 Department of Gynecology, People’s Hospital of Gengma Dai and Wa Autonomous County, Lincang 677599, China; luochengfeng@51mch.com

**Keywords:** ERBB3, endometrial cancer, cell proliferation, cell migration, the Ras-ERK/MAPK signaling pathway

## Abstract

Background: Endometrial cancer is a common gynecological malignancy, and elucidating its molecular basis may provide new clues for targeted intervention. This study investigated the role of ERBB3 in endometrial cancer cells and explored whether Ras-ERK/MAPK signaling is involved in ERBB3-mediated regulation. Methods: Bioinformatic screening was performed using public databases to identify candidate genes associated with endometrial cancer. ERBB3 was selected for further analysis. ERBB3 expression was evaluated in public datasets and examined in endometrial cancer cells. Loss-of-function experiments, rescue assays, Western blotting, and co-immunoprecipitation were used to assess the biological function and potential mechanism of ERBB3. Results: ERBB3 knockdown significantly inhibited proliferation, migration, and invasion, and promoted apoptosis in Ishikawa and RL95-2 cells. These changes were accompanied by decreased Ras-ERK/MAPK signaling. Moreover, Ras overexpression partially reversed the effects induced by ERBB3 knockdown. Co-immunoprecipitation suggested a molecular association between ERBB3 and Ras within a protein complex, but did not demonstrate direct physical binding. Conclusions: ERBB3 appears to promote malignant behaviors in endometrial cancer cells, and the Ras-ERK/MAPK pathway may be one of the downstream mechanisms involved. Further validation in additional experimental models and clinical samples is needed.

## Simple Summary

Endometrial cancer is one of the most common gynecological malignancies worldwide, with an increasing incidence and a need for novel molecular therapeutic targets. This study aimed to clarify the role and underlying mechanism of ERBB3 in endometrial cancer progression. Database analyses, including DisGeNET, GeneCards, UALCAN, and GEPIA, identified ERBB3 as a potential target gene and showed that ERBB3 is upregulated in endometrial cancer tissues and cell lines, especially Ishikawa and RL95-2 cells. In vitro experiments showed that ERBB3 knockdown inhibited cell viability, proliferation, migration, and invasion, while promoting apoptosis in Ishikawa and RL95-2 cells. Mechanistically, ERBB3 knockdown was associated with reduced Ras and phosphorylated ERK levels and increased phosphorylated p38 levels, and these molecular changes, together with the functional alterations, were partially reversed by Ras overexpression. Collectively, these findings suggest that ERBB3 contributes to malignant behaviors of endometrial cancer cells with involvement of the Ras-ERK/MAPK pathway, and that ERBB3 may have potential biological and therapeutic relevance in endometrial cancer, although further validation is required.

## 1. Introduction

Among the global female populace, endometrial cancer ranks as the fourth most prevalent malignancy [1]. In 2020, it was estimated that in the United States, there were 65,620 new cases and 12,590 subsequent deaths [2]. In China, the incidence of endometrial cancer is escalating, showing a significant trend towards diagnosis at younger ages [3]. In 2015, an estimated total of 53,600 cases of endometrial cancer were recorded, demonstrating a crude incidence rate of 7.74 per 100,000 individuals and resulting in 10,700 fatalities in China [4]. Included in the common symptoms of endometrial cancer are abnormal vaginal bleeding, pelvic pain, pain during intercourse, and others [5]. Age, hormonal factors, reproductive factors, family history, diabetes, and endometrial hyperplasia are common risk factors of endometrial cancer [6]. In the last decade, our comprehension of endometrial cancer has undergone a significant evolution, transitioning from the simple two-tiered clinicopathologic classification of type I and type II endometrial cancer to the delineation of four distinct molecular subtypes unveiled through the cancer genome atlas (TCGA) in 2013 [7]. The prognosis of early-stage endometrial cancer patients is relatively favorable, while those with advanced-stage disease exhibit poorer treatment responses and prognosis [8]. The overall 5-year survival of endometrial cancer patients is around 80% [9]. Prognosis in endometrial cancer is influenced by various factors such as tumor grade, stage, histological subtypes, lymph node metastasis, and myometrial invasion [10]. However, the pathogenesis of endometrial cancer is still unclear.

ERBB3, recognized as HER3, is a gene responsible for encoding a protein named epidermal growth factor receptor 3 [11]. Part of the receptor tyrosine kinase family associated with epidermal growth factor receptors (EGFRs), ERBB3 performs vital functions in cellular proliferation, growth, differentiation, and viability [12]. Aberrant activation of ERBB3 signaling, often through overexpression or mutations in the gene, can promote cancer cell growth, survival, and metastasis [13]. Elevated levels of ERBB3 expression have been noted across various cancer categories, such as breast cancer [14], ovarian cancer [15], gastric cancer [16], colorectal cancer [17], and others. As an avenue brimming with potential, gene therapy involves the introduction of genetic material into a patient’s cells to address genetic anomalies or deliver therapeutic advantages in the treatment of genetic disorders and specific ailments [18]. Within the expansive realm of gene therapy lies the pledge of numerous groundbreaking treatments poised to play a vital role in averting cancer-related fatalities [19]. Previous studies showed that ERBB3 could affect the development of cancers by activating or inhibiting cellular signaling pathways [20]. ERBB3-initiated signaling plays a key role in the development of cancer drug resistance, and can overcome the resistance and enhance efficacy of cancer therapeutics [21]. ERBB3 binding proteins, which exert a negative regulatory effect on ERBB3 protein levels and its capacity to convey proliferative signals, play a role in the progression of breast cancer and the development of resistance to treatment [22]. Nonetheless, its precise functional and mechanistic role in endometrial cancer remains to be further clarified.

The Ras-ERK/MAPK signaling pathway regulates a wide range of cellular processes in eukaryotic organisms, including proliferation, differentiation, homeostasis, and survival. Sustained activation of the Ras-ERK/MAPK pathway is frequently associated with abnormal cell growth and tumorigenesis [23]. In endometrial cancer, activation of ERK1/2 has also been reported, and phosphorylated ERK1/2 expression was shown to occur independently of KRAS or BRAF status and to be associated with prognosis [24]. It has been reported that the continuous renewal of epidermal cells partly depends on persistent proliferative and survival signals transmitted through the Ras-ERK/MAPK pathway [25]. Pharmacological inhibition of this pathway by TLN-4601 suppresses proliferation and induces apoptosis in pancreatic carcinoma cells [26]. In acute myeloid leukemia, NPM1 mutations enhance cellular invasiveness by upregulating matrix metalloproteinases through activation of the Ras-ERK/MAPK pathway [27]. In the context of endometrial cancer, Asparanin A has been shown to inhibit cell migration and invasion through modulation of the Ras-ERK/MAPK pathway [28]. These findings suggest that Ras-ERK/MAPK signaling is involved in tumor progression, including in endometrial cancer. However, whether ERBB3-mediated regulation in endometrial cancer involves this pathway remains to be determined.

Accordingly, the present study investigated the functional significance of ERBB3 in endometrial cancer and explored whether Ras-ERK/MAPK signaling might be involved in ERBB3-mediated regulation. By combining bioinformatic screening, loss-of-function experiments, rescue assays, and molecular analyses, we sought to clarify whether ERBB3 contributes to the malignant phenotype of endometrial cancer cells and whether the Ras-ERK/MAPK pathway may represent one of its downstream mechanisms.

## 2. Material and Methods

### 2.1. Reagents

Antibodies against ERBB3 (12708S), Ras (3965S), phospho-ERK (5726S), ERK (4695S), phospho-p38 (4511S), p38 (9212S), Ki-67 (9129S), and GAPDH (2118S) were purchased from Cell Signaling Technology (Beverly, MA, USA). The Cell Counting Kit-8 (CCK-8; C0037), 5-ethynyl-2′-deoxyuridine (EdU) Cell Proliferation Kit (C0071S), and Terminal deoxynucleotidyl transferase dUTP nick end labeling (TUNEL) Apoptosis Assay Kit (C1088) were obtained from Beyotime (Beijing, China).

### 2.2. Cell Culture and Cell Transfection

The hEEC, RL95-2, Ishikawa, HEC1B, and AN3CA cell lines, procured from the American Type Culture Collection (ATCC; Manassas, VA, USA), and cultured in the appropriate recommended medium, DMEM/F12 or RPMI-1640, supplemented with 10% fetal bovine serum and 1% penicillin-streptomycin from Merck (Darmstadt, Germany), along with 1% penicillin-streptomycin also from the same supplier. Cells exhibiting active growth and a trypan blue exclusion rate surpassing 95% were selected for subsequent assays. The utilized sequences were as follows: siRNA-ERBB3, 5′-CCGGUGGCUCUAAUGCCGA-3′; siRNA-negative control (siRNA-NC), 5′-UUCUCCGAACGUGUCACGU-3′; pc-Ras, 5′-ATGGCGGAGGAGCGCCAT-3′; pc-negative control (pc-NC), 5′-GGAGGCUCUACUGCCGA-3′. These sequences were acquired from GenePharma (Shanghai, China). A siRNA transfection protocol entailed 20 nM siRNA and Lipofectamine^®^ 2000 from Thermo Fisher Scientific, Inc. (Waltham, MA, USA), as per the manufacturer’s directives. Following assorted treatments administered within a 24 h timeframe, cells were harvested for functional assessments, while RNA and protein were extracted for ensuing qRT-PCR and Western blot analyses.

### 2.3. Disease Gene Targets

Disease-associated genes related to endometrial cancer were retrieved from the DisGeNET (https://www.disgenet.org/home/ accessed on 27 January 2025) and GeneCards (https://www.genecards.org/ accessed on 28 January 2025) databases. The search term used was “endometrial cancer”, and the species was restricted to *Homo sapiens*.

### 2.4. Gene Expression Analysis

ERBB3 expression in endometrial cancer was analyzed using the GEPIA database (http://gepia.cancer-pku.cn/detail.php accessed on 28 January 2025), an online platform based on TCGA and GTEx datasets. The expression levels of ERBB3 were compared between endometrial cancer tissues (*n* = 174) and normal endometrial tissues (*n* = 91). A value of *p* < 0.05 was considered statistically significant.

### 2.5. Cell Viability

Under the circumstances of 37 °C and 5% CO_2_, cell cultivation in 96-well plates endured for a period of 24 h. Cell viability was assessed using the CCK-8 assay, the CCK-8 kit (C0037, Beyotime, Beijing, China) was employed, with measurement of the optical density (OD) at 450 nm achieved through a microplate reader. The procedures were carried out in 8 sets running concurrently, with each set undergoing 3 replicates.

### 2.6. EdU Assay

Employing the EdU assay kit (C0071S, Beyotime, Beijing, China), cellular proliferation was assessed. Cells were planted, processed, and exposed to the EdU buffer. After undergoing fixation and permeabilization, cellular staining with DAPI took place. Utilizing ImageJ software (V1.8.0, NIH, Bethesda, MD, USA), fluorescence images were captured and analyzed to ascertain the proliferation rate of the cells.

### 2.7. Immunofluorescence

Cell fixation was carried out using pre-chilled acetone. Subsequently, cell washing was performed thrice with PBS. Permeabilization of cells took place with Triton X-100 (0.1%), followed by treatment with BSA/PBS (1%). Next, the cells were exposed to the primary antibody targeting Ki-67 (diluted 1:200) and left to incubate overnight at 4 °C. Subsequent to this, the cells underwent a triple washing with PBS. After washing with PBS, the cells were incubated with the corresponding fluorophore-conjugated secondary antibody for 1 h at room temperature in the dark. DAPI staining was employed for nucleus visualization. Imaging was conducted using a Zeiss inverted microscope (Zeiss, Oberkochen, Germany).

### 2.8. Transwell Experiment

Migration analysis was carried out utilizing transwell chambers (Corning, Corning, NY, USA) equipped with 8-μm pore size. Initially, in the upper segment of the chamber, 8 × 10^5^ cells were seeded in serum-deficient medium. A lower chamber was infused with medium comprising 10% FBS serving as the chemoattractant. The incubation at 37 °C persisted for 24 h. Subsequently, removable permeable inserts were extracted, and the migratory cells were fixed, stained, and quantified (at a magnification of ×200). These procedures were iterated at least thrice. The visualization of cellular images was assessed utilizing the ImageJ software (V1.8.0, NIH, Bethesda, MD, USA). In regard to invasion assessment, similar to the previous migration trials, both negative controls and transfected cells were subjected to exposure within 24-well BD Biocoat Matrigel Invasion Chambers (BD Biosciences, Franklin Lakes, NJ, USA).

### 2.9. TUNEL Assay

Cell death programming assessment was conducted using the apoptosis detection kit (C1088, Beyotime, Beijing, China) employing the TUNEL technique. Cells were cultured and positioned on chamber slides (Becton Dickinson, Franklin Lakes, NJ, USA) for on-site apoptosis evaluation. The marking of DNA fragments at the free 3′-OH DNA termini and DNA strand ruptures in apoptotic cells was performed. The existence of integrated fluorescein was identified using alkaline phosphatase-bound anti-fluorescein antibodies. Following the substrate reaction, stained cells were observed under light microscopy (Cx43, Olympus, Hachioji-shi, Tokyo, Japan).

### 2.10. Quantitative Real-Time PCR (qRT-PCR)

Total RNA was extracted from cells using TRIzol reagent (Beyotime, Beijing, China) according to the manufacturer’s instructions. The extracted RNA was reverse-transcribed into cDNA using a cDNA synthesis kit (Beyotime, Beijing, China). Quantitative real-time PCR was then performed using an ABI 7900 Real-Time PCR System (ABI, Los Angeles, CA, USA). The primer sequences were as follows: ERBB3 (human), forward 5′-CTTCCTGCAGTGGATTCGA-3′ and reverse 5′-AGGTTGGGCAATGGTAGAG-3′; Ras (human), forward 5′-GAGTGCCTTGACGATACAG-3′ and reverse 5′-TGCTTCCTGTAGGAATCCTC-3′; GAPDH (human), forward 5′-TCAAGATCATCAGCAATGCC-3′ and reverse 5′-CGATACCAAAGTTGTCATGGA-3′. GAPDH was used as the internal control, and relative mRNA expression levels were calculated using the 2^−ΔΔCt^ method.

### 2.11. Western Blot

Total protein was extracted from cells using lysis buffer, and protein concentrations were determined using a BCA protein assay kit. Equal amounts of protein were separated by SDS-PAGE and transferred onto polyvinylidene fluoride (PVDF) membranes. The membranes were blocked with 5% skim milk and then incubated overnight at 4 °C with primary antibodies against ERBB3, Ras, p-ERK, ERK, p-p38, p38, and GAPDH (all diluted 1:1000). After washing, the membranes were incubated with the corresponding HRP-conjugated secondary antibody at room temperature for 1 h. Protein bands were visualized using an enhanced chemiluminescence (ECL) detection system. GAPDH was used as the internal loading control. Band intensities were quantified by densitometric analysis using ImageJ software (version 1.8.0; NIH, Bethesda, MD, USA).

### 2.12. Co-Immunoprecipitation (Co-IP)

To further investigate the molecular association between ERBB3 and Ras in endometrial cancer cells, co-immunoprecipitation (Co-IP) assays were performed. Cells were lysed in IP lysis buffer supplemented with protease inhibitors, and the lysates were centrifuged to collect the supernatants. Equal amounts of total protein were incubated overnight at 4 °C with an anti-ERBB3 antibody, while normal IgG was used as a negative control. The immune complexes were then captured using Protein A/G agarose beads, washed several times with lysis buffer, and analyzed by Western blotting with the indicated antibodies. Input lysates were used as positive controls.

### 2.13. Statistical Analysis

All experiments were independently repeated at least three times. Data are presented as mean ± SD. Statistical analyses were performed using GraphPad Prism 8.0.2(263). Comparisons between two groups were performed using Student’s *t*-test. Comparisons among multiple groups were analyzed using one-way ANOVA followed by Tukey’s multiple comparisons test. For CCK-8 assays involving both group and time factors, two-way ANOVA followed by Tukey’s multiple comparisons test was used. A *p* value < 0.05 was considered statistically significant.

## 3. Results

### 3.1. ERBB3 Is Highly Expressed in Endometrial Cancer Tissues and Cells

**Figure 1:**
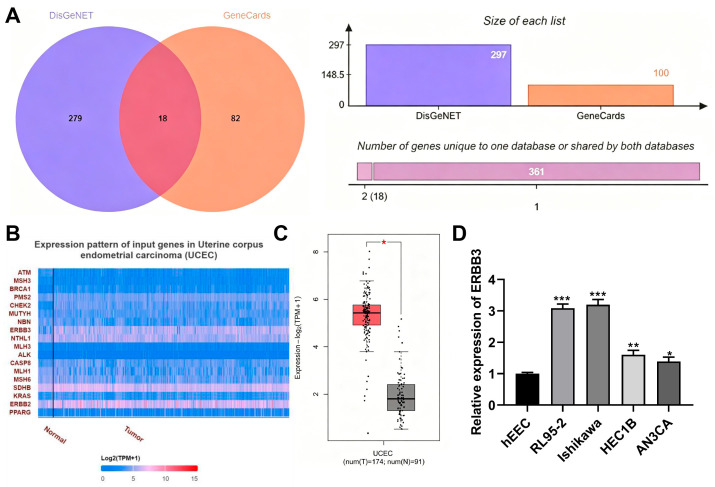
The expression of ERBB3 in endometrial cancer. (**A**) Identification of targeted genes of endometrial cancer by DisGeNET and GeneCards databases. (**B**) The expression of targeted genes in endometrial cancer tissues analyzed by UALCAN. (**C**) ERBB3 expression in endometrial cancer patients analyzed by GEPIA. (**D**) ERBB3 expression in endometrial cancer cell lines detected by Real-time PCR. * *p* < 0.05, ** *p* < 0.01, *** *p* < 0.001.

By intersecting endometrial cancer-related genes retrieved from DisGeNET and GeneCards, 18 overlapping candidate genes were obtained, as shown in Figure 1A. These candidates were further examined using UALCAN and GEPIA expression analyses. ERBB3 was prioritized for subsequent validation because it was consistently upregulated in endometrial cancer tissues and is functionally related to growth factor receptor-mediated oncogenic signaling. Analysis using the UALCAN database indicated that ERBB3 was highly expressed in endometrial cancer tissues (Figure 1B). This finding was further confirmed by GEPIA, which showed that ERBB3 was significantly upregulated in patients with endometrial cancer (*p* < 0.05, Figure 1C).

We next evaluated ERBB3 expression in hEEC, RL95-2, Ishikawa, HEC1B, and AN3CA cells. qRT-PCR analysis showed that ERBB3 expression was significantly increased in RL95-2, Ishikawa, HEC1B, and AN3CA cells compared with normal human endometrial epithelial cells (hEEC) (*p* < 0.05, Figure 1D). These findings suggest that ERBB3 overexpression is a common feature of endometrial cancer cells at the transcriptional level. Among these cell lines, Ishikawa and RL95-2 cells were selected for subsequent experiments because they are widely used endometrial cancer models and provide complementary biological characteristics for in vitro functional validation.

### 3.2. Transfection Efficiency of si-ERBB3 in Ishikawa and RL95-2 Cells

**Figure 2:**
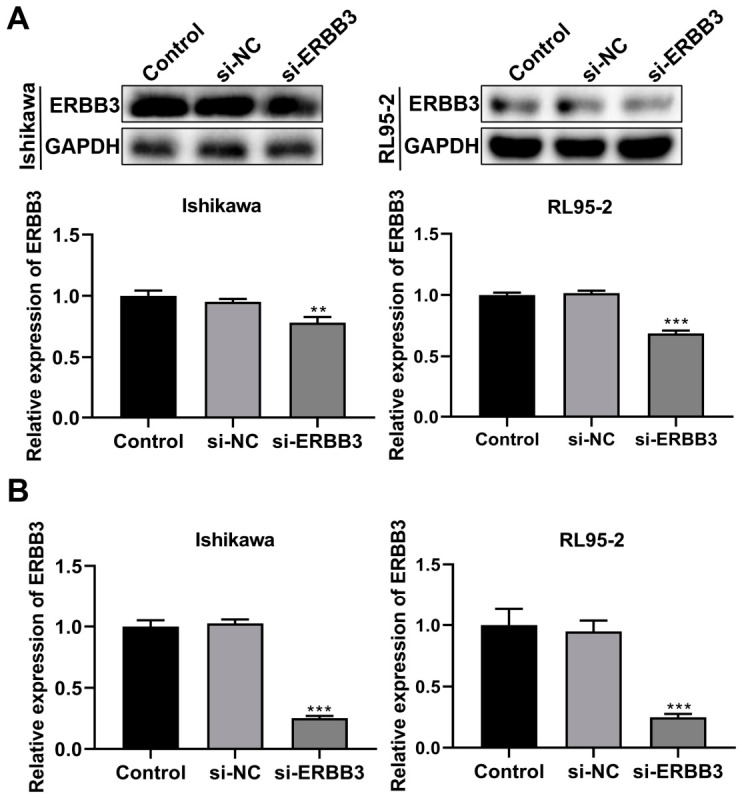
Transfection efficiency of si-ERBB3 in Ishikawa and RL95-2 cells. (**A**) The expression of ERBB3 by Western blot. The above were the representative band diagrams. (**B**) The expression of ERBB3 by Realtime PCR. ** *p* < 0.01, *** *p* < 0.001. Original western blots are presented in File S1.

To evaluate the transfection efficiency of si-ERBB3 in endometrial cancer cells, ERBB3 expression was measured by Western blotting and real-time PCR. Western blot analysis showed that ERBB3 protein levels were significantly reduced in Ishikawa (*p* < 0.01) and RL95-2 cells (*p* < 0.001, Figure 2A). Although the visual difference in protein bands was less pronounced than that observed at the mRNA level, ImageJ-based densitometric analysis confirmed that the reduction in ERBB3 protein expression was statistically significant. This discrepancy may be related to the half-life of ERBB3 protein or differences in assay sensitivity.

Consistently, real-time PCR analysis demonstrated that ERBB3 mRNA levels were markedly decreased in both cell lines following si-ERBB3 transfection (*p* < 0.001, Figure 2B). Taken together, these results confirm the successful silencing of ERBB3 in Ishikawa and RL95-2 cells.

### 3.3. Effects of Downregulation of ERBB3 on the Cell Function in Endometrial Cancer Cells

**Figure 3:**
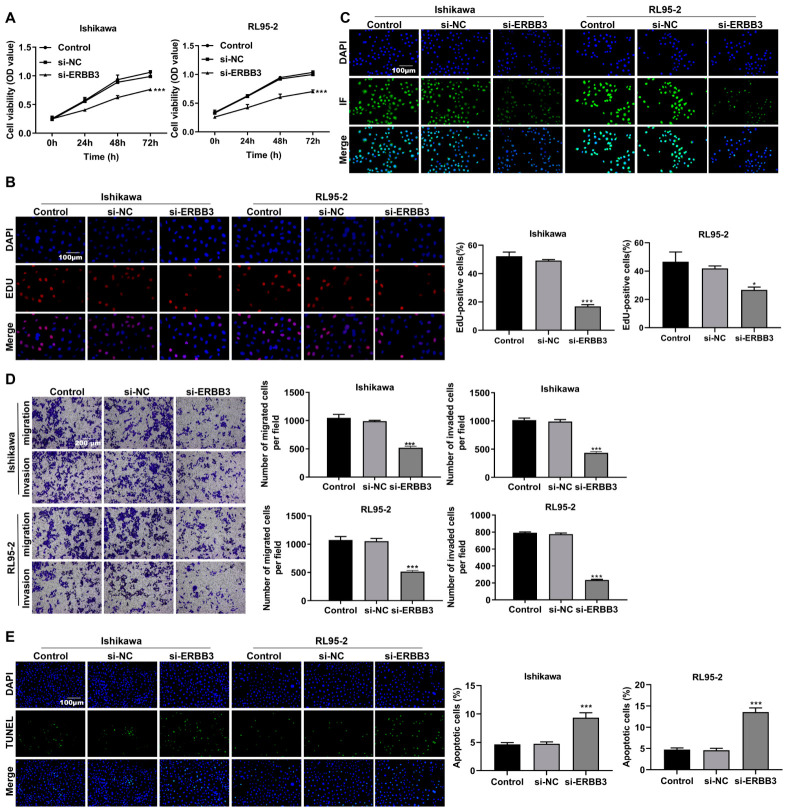
Effects of downregulation of ERBB3 on the cell function in Ishikawa and RL95-2 cells. (**A**) Cell viability by CCK-8 assay. (**B**) Cell proliferation by EdU assay. (**C**) The expression of Ki-67 by IF. (**D**) Cell migration and invasion by Transwell assay. (**E**) Cell apoptosis by TUNEL assay. * *p* < 0.05, *** *p* < 0.001. Data are presented as mean ± SD from three independent biological replicates.

Following transfection with si-ERBB3, cell viability was significantly reduced in both Ishikawa and RL95-2 cells (*p* < 0.001, Figure 3A). EdU assays further showed that cell proliferation was significantly inhibited in these two cell lines (*p* < 0.05, Figure 3B). Ki-67 is a widely used marker for assessing cell proliferation in both research and clinical settings [29]. Consistently, Ki-67 expression was significantly decreased in the si-ERBB3 group (Figure 3C).

In addition, Transwell assays showed that both migration and invasion were markedly suppressed after ERBB3 knockdown (*p* < 0.001, Figure 3D). TUNEL staining demonstrated that si-ERBB3 significantly promoted apoptosis in Ishikawa and RL95-2 cells (*p* < 0.001, Figure 3E). These data indicate that ERBB3 downregulation inhibits the malignant phenotype of endometrial cancer cells.

### 3.4. ERBB3 Downregulation Is Associated with Altered Ras-ERK/MAPK Signaling in Endometrial Cancer Cells

**Figure 4:**
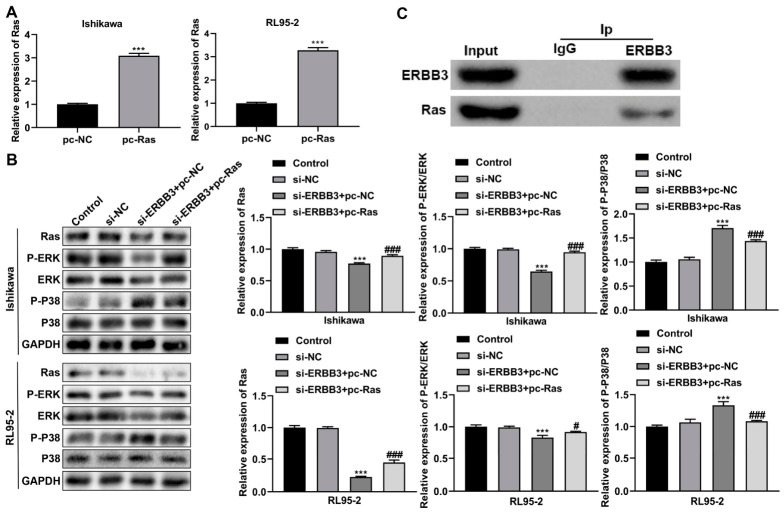
Effects of downregulation of ERBB3 on the Ras-ERK/MAPK signaling pathway and its molecular association with Ras in endometrial cancer cells. (**A**) Ras expression measured by real-time PCR. (**B**) Expression levels of Ras, P-ERK, ERK, P-P38, and P38 detected by Western blot. (**C**) Co-IP assay suggesting a molecular association between ERBB3 and Ras. Data are presented as mean ± SD from three independent biological replicates. *** *p* < 0.001; vs. si-ERBB3 + pc-NC, ^#^ *p* < 0.05, ^###^ *p* < 0.001. Original western blots are presented in File S1.

To explore whether Ras signaling is involved in ERBB3-mediated regulation, the transfection efficiency of pc-Ras was first assessed in Ishikawa and RL95-2 cells by real-time PCR. Following transfection with pc-Ras, Ras mRNA expression was significantly increased in both cell lines (*p* < 0.001, Figure 4A), confirming successful Ras overexpression.

Western blot analysis showed that, compared with the control group, Ras and p-ERK protein levels were decreased in the si-ERBB3 + pc-NC group, whereas p-p38 exhibited an opposite trend. Specifically, p-p38 levels remained low in the control groups, increased markedly after ERBB3 knockdown, and were partially reduced following Ras overexpression. In addition, Ras overexpression partially restored the decreased Ras and p-ERK expression observed after ERBB3 silencing (*p* < 0.05, Figure 4B). These findings suggest that ERBB3 downregulation is associated with suppression of Ras-ERK signaling and concurrent activation of p38-related stress signaling.

To further investigate the relationship between ERBB3 and Ras in endometrial cancer cells, co-immunoprecipitation (Co-IP) assays were performed. As shown in Figure 4C, Ras was detected in ERBB3 immunoprecipitates, whereas no obvious Ras signal was observed in the IgG control group. ERBB3 was efficiently precipitated in the anti-ERBB3 group, confirming the validity of the immunoprecipitation assay. These results suggest that ERBB3 and Ras may exist within the same protein complex in endometrial cancer cells. However, the present Co-IP data do not establish direct physical binding between ERBB3 and Ras and should therefore be interpreted as evidence of molecular association rather than direct interaction.

Taken together, these findings support the possibility that ERBB3 regulates malignant behaviors of endometrial cancer cells with involvement of the Ras-ERK/MAPK signaling pathway.

### 3.5. Ras Overexpression Partially Reverses the Effects Induced by ERBB3 Downregulation in Endometrial Cancer Cells

**Figure 5:**
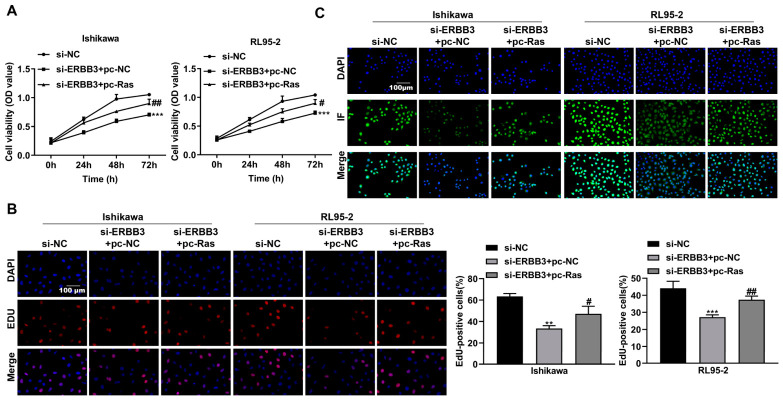

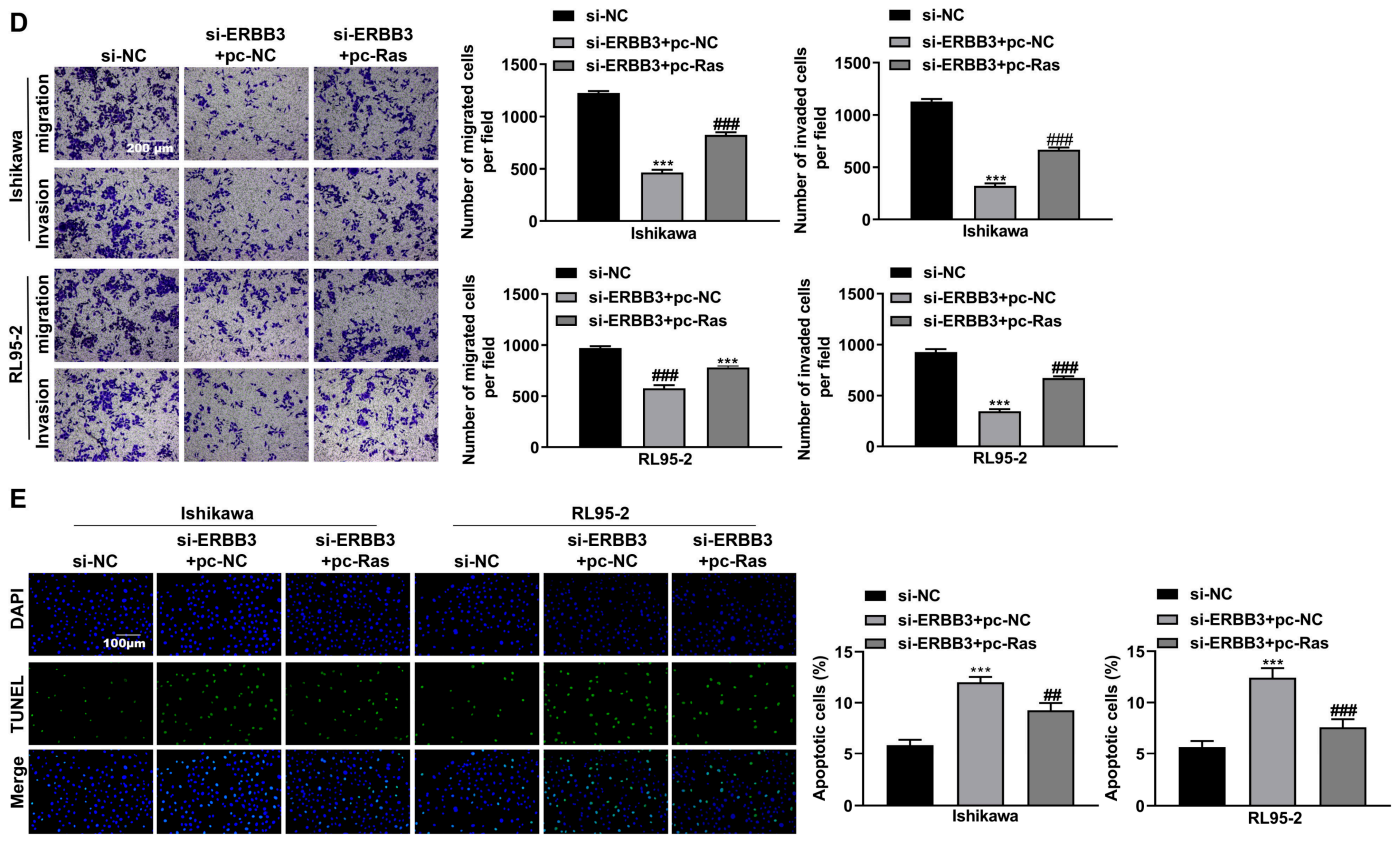
Effects of ERBB3 downregulation on endometrial cancer cell behaviors with involvement of the Ras-ERK/MAPK signaling pathway. (**A**) Cell viability assessed by CCK-8 assay. (**B**) Cell proliferation by EdU assay. (**C**) The expression of Ki-67 by IF. (**D**) Cell migration and invasion by Transwell assay. (**E**) Cell apoptosis by TUNEL assay. vs. si-NC, ** *p* < 0.01, *** *p* < 0.001. vs. si-ERBB3 + pc-NC, ^#^ *p* < 0.05, ^##^ *p* < 0.01, ^###^ *p* < 0.001. Data are presented as mean ± SD from three independent biological replicates.

To further examine whether Ras-ERK/MAPK signaling is involved in the biological effects of ERBB3 downregulation, rescue experiments were performed in Ishikawa and RL95-2 cells. After co-transfection with si-ERBB3 and pc-NC, cell viability was significantly reduced in both cell lines (*p* < 0.001), whereas Ras overexpression partially restored cell viability (*p* < 0.05, Figure 5A).

Similarly, EdU assays showed that cell proliferation was significantly decreased in the si-ERBB3 + pc-NC group (*p* < 0.01), and this inhibitory effect was partially reversed by pc-Ras (*p* < 0.05, Figure 5B). Consistent with these findings, Ki-67 expression was markedly reduced in the si-ERBB3 + pc-NC group but increased after Ras overexpression (Figure 5C).

Moreover, migration and invasion were significantly impaired in cells transfected with si-ERBB3 + pc-NC (*p* < 0.001), whereas these effects were partially attenuated by pc-Ras (*p* < 0.001, Figure 5D). TUNEL assays showed that apoptosis was significantly increased after transfection with si-ERBB3 + pc-NC in both Ishikawa and RL95-2 cells (*p* < 0.001), while Ras overexpression partially reduced apoptosis (*p* < 0.01, Figure 5E).

Overall, these results indicate that Ras overexpression partially reverses the phenotypic changes induced by ERBB3 knockdown, supporting the involvement of Ras-ERK/MAPK signaling in ERBB3-mediated regulation of endometrial cancer cells.

## 4. Discussion

Endometrial cancer is one of the most common malignant tumors of the female reproductive system, and its progression is closely associated with dysregulation of cell proliferation, migration, invasion, and apoptosis [30]. Although substantial advances have been made in the understanding and treatment of endometrial cancer, the molecular mechanisms underlying its progression remain incompletely understood. In the present study, we investigated the biological role of ERBB3 in endometrial cancer and explored the possible involvement of the Ras-ERK/MAPK signaling pathway. Our findings showed that downregulation of ERBB3 significantly inhibited the malignant behaviors of endometrial cancer cells and promoted apoptosis. In addition, rescue experiments supported the functional involvement of Ras signaling in ERBB3-mediated regulation.

ERBB3, a member of the epidermal growth factor receptor family, has been reported to participate in the progression of multiple human malignancies. Increasing evidence suggests that aberrant ERBB3 activation contributes to tumor cell proliferation, survival, metastasis, and therapeutic resistance [31]. In agreement with these observations, our results demonstrated that silencing ERBB3 in Ishikawa and RL95-2 cells markedly reduced cell viability and proliferation, as evidenced by CCK-8, EdU, and Ki-67 results [32]. Meanwhile, ERBB3 knockdown significantly suppressed cell migration and invasion while promoting apoptosis. These findings suggest that ERBB3 contributes to the malignant phenotype of endometrial cancer cells and that its upregulation may be associated with the aggressive biological behavior of this malignancy.

To further explore the underlying mechanism, we focused on the Ras-ERK/MAPK signaling pathway, a classical pathway involved in regulating cell growth, survival, differentiation, and motility. Dysregulation of this pathway has been widely implicated in the initiation and progression of many cancers, including endometrial cancer. In the present study, ERBB3 downregulation reduced Ras expression and decreased the phosphorylation level of ERK, whereas p-p38 showed an opposite trend. Specifically, p-p38 remained low in the control groups, increased markedly after ERBB3 knockdown, and was partially reduced after Ras overexpression. These findings suggest that ERBB3 downregulation is associated with suppression of Ras-ERK signaling while being accompanied by activation of p38-related stress signaling. Because p38 is frequently linked to cellular stress and apoptosis-related responses, the increased p-p38 level observed after ERBB3 silencing may reflect stress signaling activation rather than a pro-malignant effect. Nevertheless, the precise biological significance of this alteration requires further investigation. Overall, these data support the involvement of Ras-ERK/MAPK signaling in ERBB3-mediated regulation [33].

Importantly, the rescue experiments further strengthened this mechanistic interpretation. After co-transfection with si-ERBB3 and pc-Ras, the inhibitory effects of ERBB3 knockdown on cell viability, proliferation, migration, and invasion were partially reversed, whereas the pro-apoptotic effect was attenuated. These results suggest that Ras signaling is involved in the malignant phenotypes regulated by ERBB3 [34]. However, the partial rescue effect induced by Ras overexpression indicates that Ras-ERK/MAPK signaling is likely an important downstream mechanism, but not necessarily the only pathway through which ERBB3 exerts its biological effects in endometrial cancer cells [35].

To further examine the relationship between ERBB3 and Ras, co-immunoprecipitation assays were performed. Ras was detected in ERBB3 immunoprecipitates, whereas no obvious Ras signal was observed in the IgG control group, suggesting that ERBB3 and Ras may exist within the same protein complex in endometrial cancer cells. This evidence supports a molecular association between ERBB3 and Ras and is consistent with the rescue and signaling results. However, it should be noted that Co-IP mainly indicates that two proteins are present in the same complex or are molecularly associated, rather than definitively proving direct physical binding. Previous studies have shown that active Ras can physically associate with downstream signaling partners, including mSIN1, a component of mTORC2, supporting the biological plausibility of Ras-related protein interactions in signaling networks [36]. Therefore, additional studies using reciprocal Co-IP, pull-down assays, proximity ligation assays, or domain-mapping strategies are required to further define the precise molecular relationship between ERBB3 and Ras.

Despite these findings, several limitations of this study should be acknowledged. First, only a single siRNA sequence was used to silence ERBB3, and off-target effects cannot be completely excluded. Second, the present work was mainly performed in two endometrial cancer cell lines, and additional cell models would further strengthen the generalizability of the conclusions. Third, in vivo experiments were not included, and therefore the role of ERBB3 in tumor growth and metastasis requires further validation in animal models. Fourth, although the current results support the involvement of the Ras-ERK/MAPK pathway, other downstream pathways may also contribute to ERBB3-mediated regulation and remain to be explored. Finally, the clinical significance of ERBB3 expression in endometrial cancer tissues and its association with patient prognosis were not evaluated in this study and deserve further investigation.

**Figure 6:**
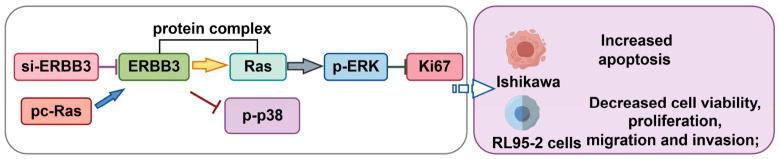
Schematic illustration of the proposed mechanism by which ERBB3 regulates malignant behaviors in endometrial cancer cells. The schematic indicates that ERBB3 may regulate proliferation, migration, invasion, and apoptosis in endometrial cancer cells with involvement of Ras-ERK/MAPK signaling. The model is based on the current experimental findings and should be interpreted as a proposed mechanism requiring further validation.

In summary, the present study suggests that ERBB3 contributes to the malignant progression of endometrial cancer cells. Downregulation of ERBB3 inhibited proliferation, migration, and invasion, while promoting apoptosis. These effects were associated with altered Ras-ERK/MAPK signaling, and Ras overexpression partially rescued the phenotype induced by ERBB3 knockdown. In addition, the Co-IP assay provided further evidence supporting a molecular association between ERBB3 and Ras. Collectively, these findings support the involvement of ERBB3 in endometrial cancer progression and provide additional insight into the molecular mechanisms underlying this disease, while further studies are needed to confirm its biological and therapeutic significance [37]. The schematic diagram illustrating the proposed mechanism of ERBB3-mediated regulation in endometrial cancer cells is presented in Figure 6.

## 5. Conclusions

In summary, this study demonstrates that ERBB3 functions as an oncogenic driver in endometrial cancer. ERBB3 is significantly upregulated in endometrial cancer tissues and cell lines, and its knockdown markedly suppresses cell proliferation, migration, and invasion while inducing apoptosis. Mechanistically, ERBB3 exerts its pro-malignant effects predominantly through activation of the Ras-ERK/MAPK signaling pathway, as evidenced by reduced Ras and phosphorylated ERK levels upon ERBB3 silencing and partial phenotypic rescue by Ras overexpression. Collectively, these findings highlight ERBB3 as a promising therapeutic target for endometrial cancer and provide novel insights into the molecular mechanism underlying its progression.

## Data Availability

The authors declare that the data supporting the findings of this study are available within the article and its Supplementary Materials files. All relevant data are available from the authors upon request.

## Supplementary Materials

The following supporting information can be downloaded at: https://www.mdpi.com/article/10.3390/cancers18111765/s1, File S1: Original blots presented in the manuscript.
