# Supplementary material for: ERBB3 Promotes Malignant Behaviors of Endometrial Cancer Cells with Involvement of the Ras-ERK/MAPK Signaling Pathway
Source: Cancers (Basel). 2026 May 28;18(11):1765. doi: 10.3390/cancers18111765 (PMC13255631; doi:10.3390/cancers18111765)
Supplement: Supplementary file 1 [file cancers-18-01765-s001.zip › cancers-4281439-supplementary.pdf]

Original blots presented in the manuscript

Source Data Figure 2

Figure.2A

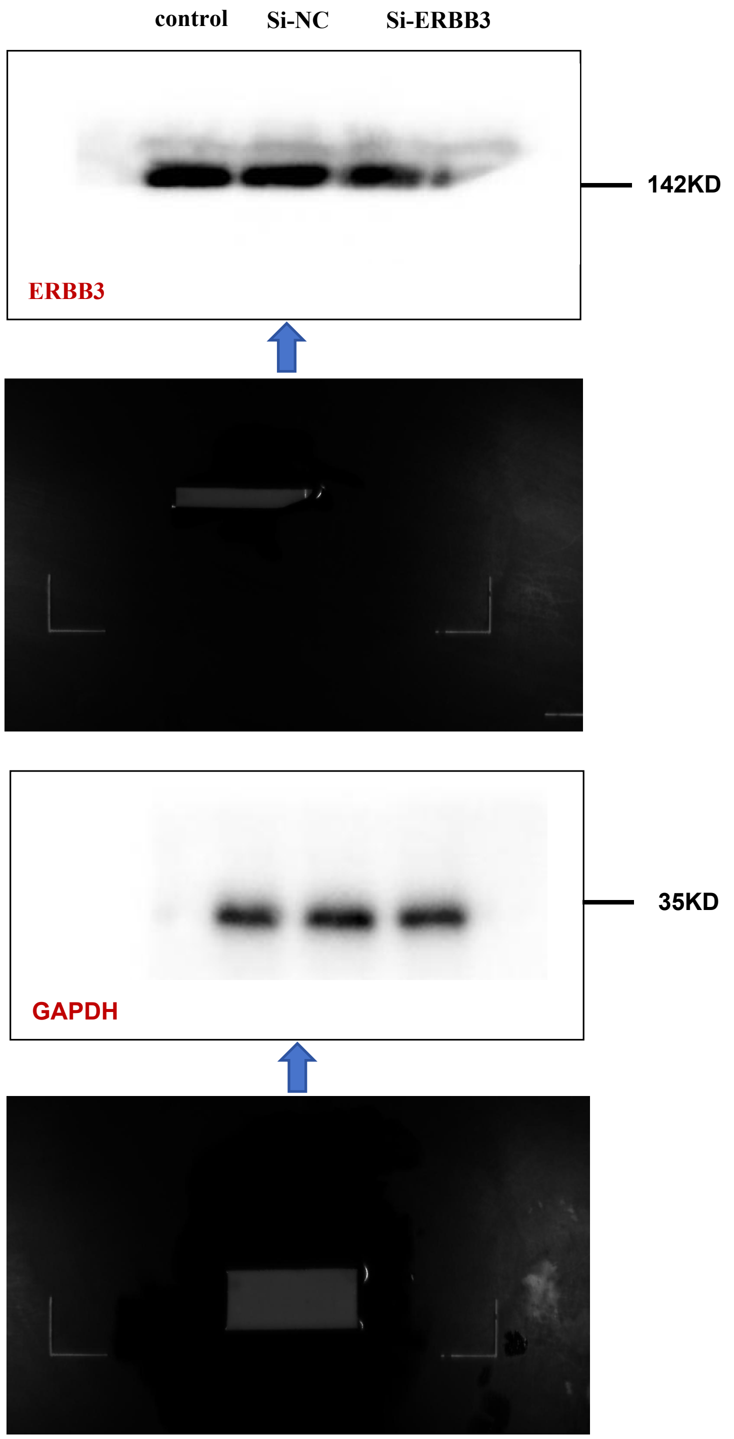

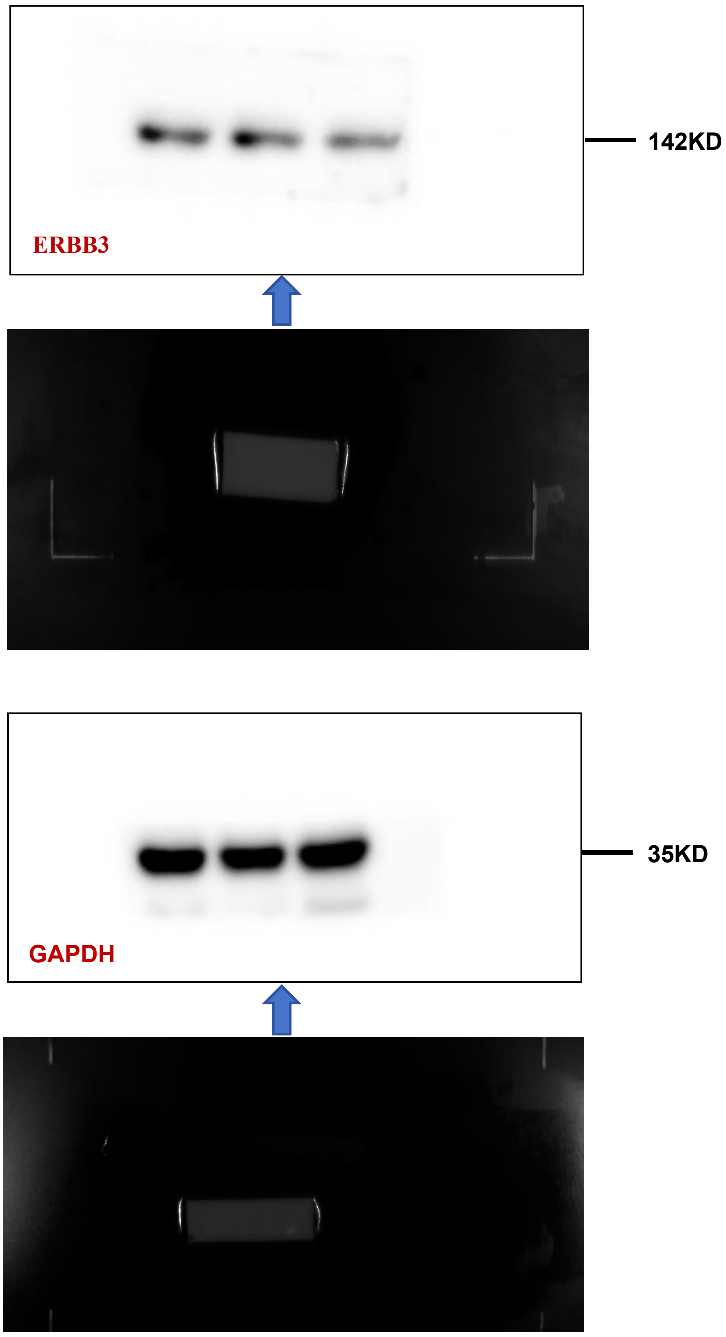
ERBB3
142KD
GAPDH
35KD

Source Data Figure 4

Figure.4B

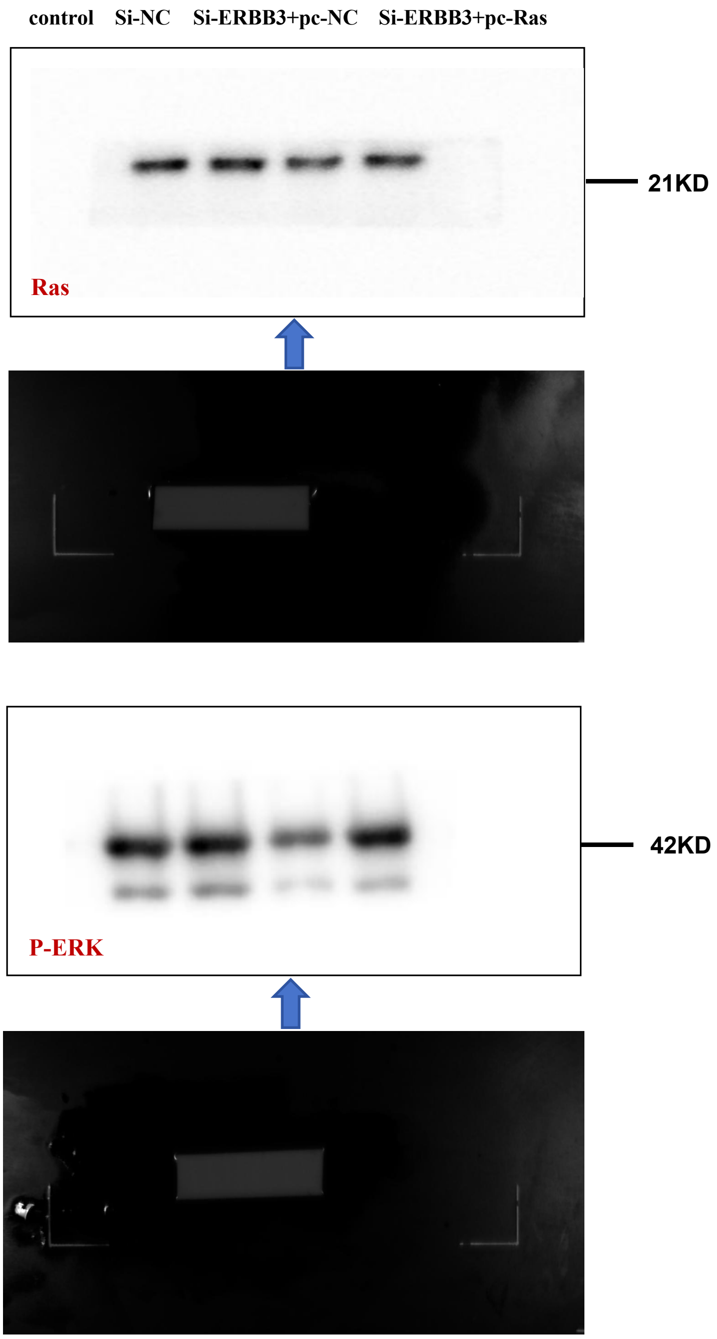

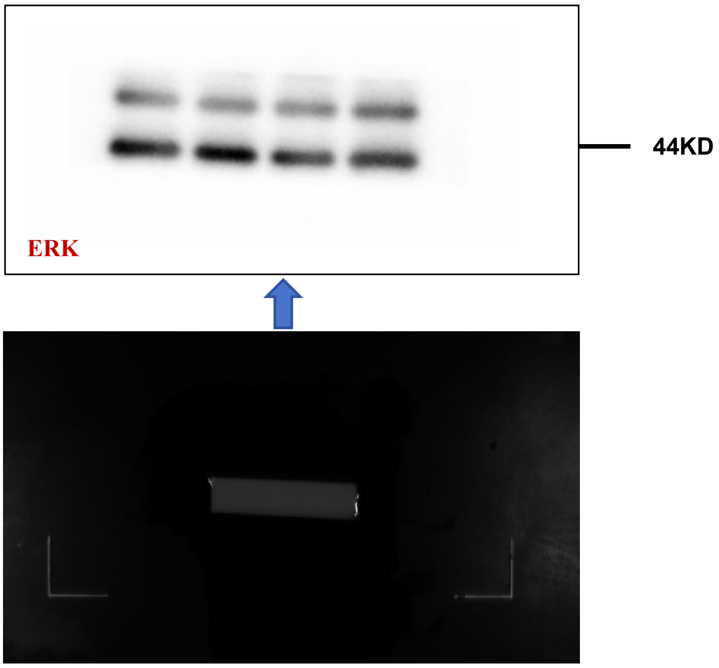
ERK
44KD

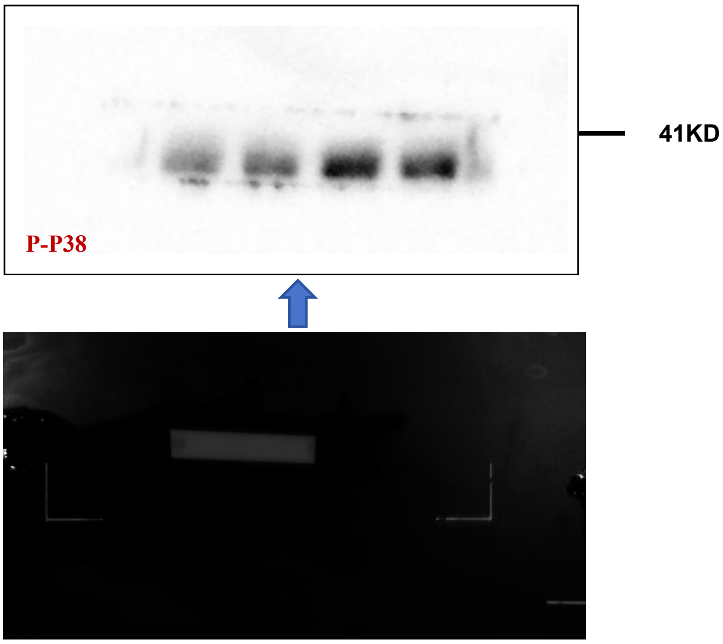
P-P38
41KD

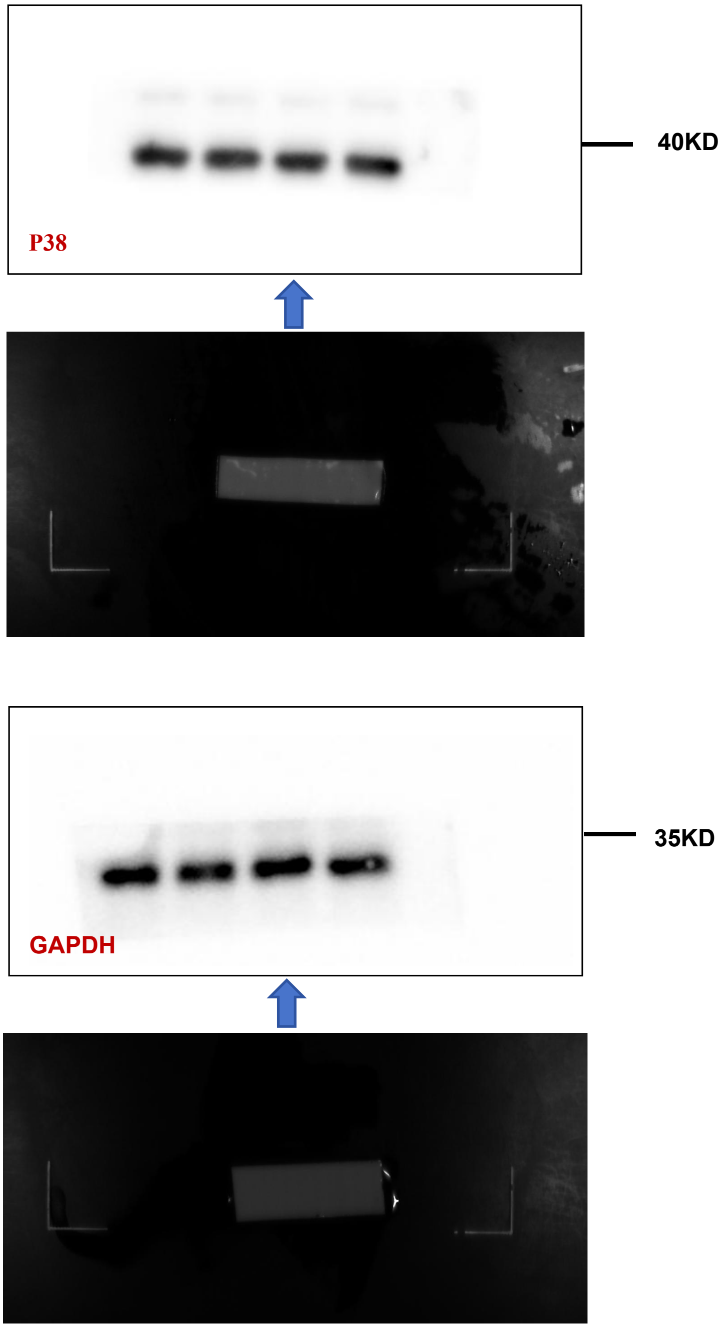
P38
40KD
GAPDH
35KD

Figure.4B

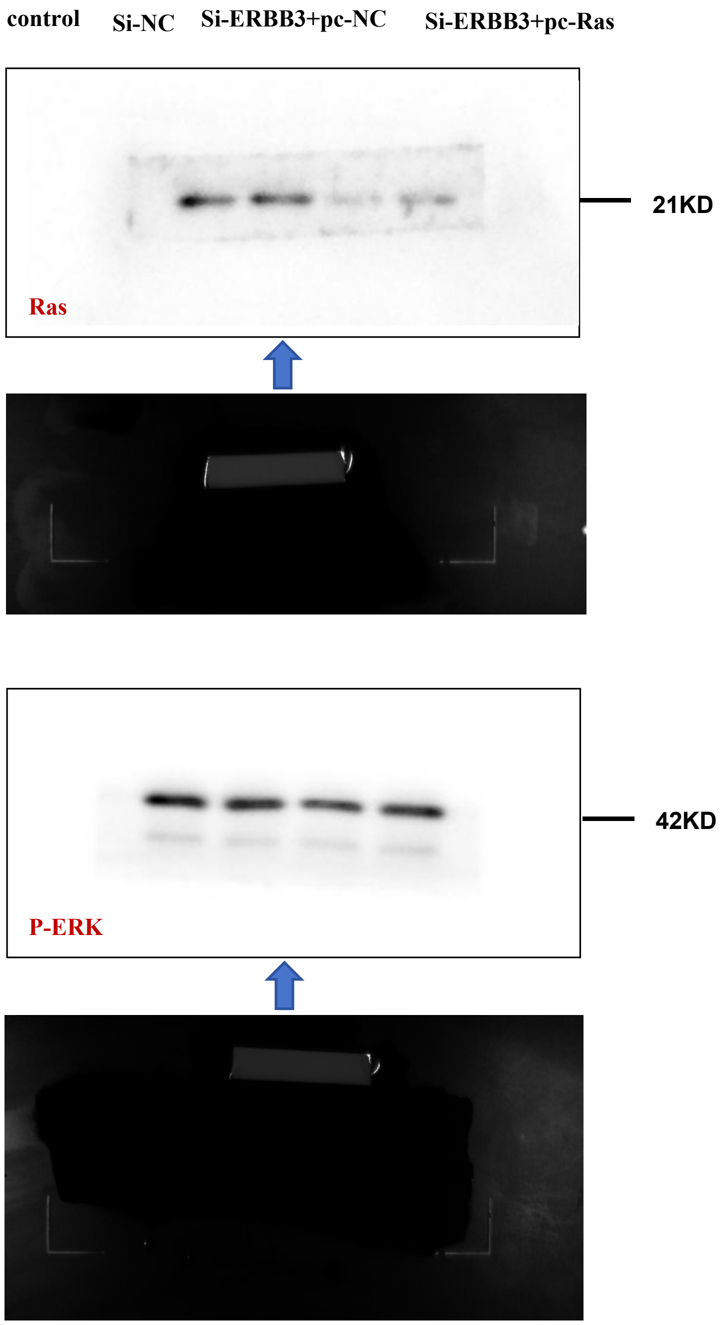

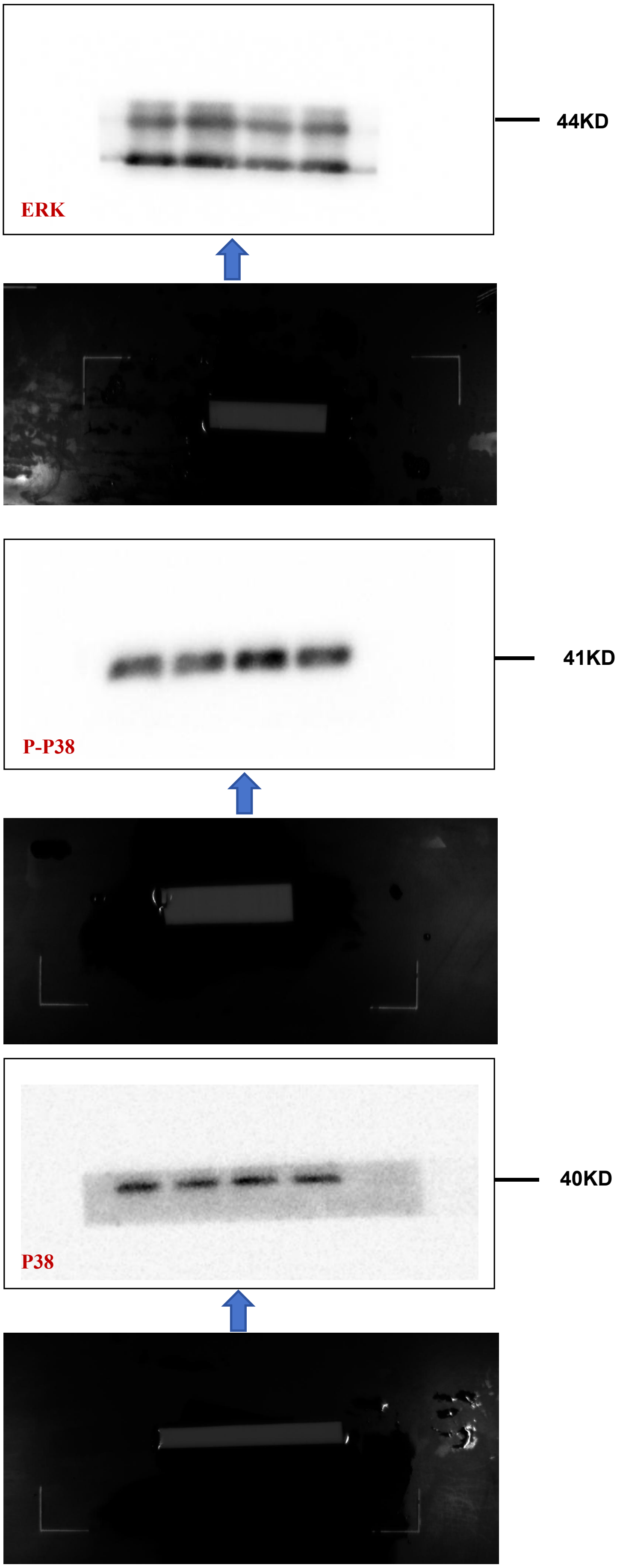
44KD
ERK
41KD
P-P38
40KD
P38

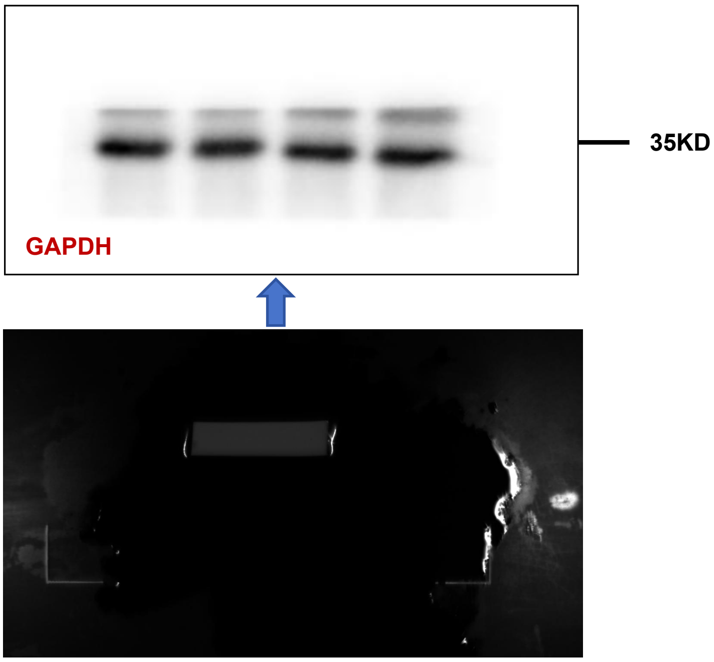
35KD
GAPDH
